# Genetic Diversity and Phenotypic Variation in an Introgression Line Population Derived from an Interspecific Cross between *Oryza glaberrima* and *Oryza sativa*

**DOI:** 10.1371/journal.pone.0161746

**Published:** 2016-09-07

**Authors:** Caijin Chen, Wenchuang He, Tondi Yacouba Nassirou, Wei Zhou, Yilong Yin, Xilong Dong, Quanqin Rao, Han Shi, Wubin Zhao, Andrew Efisue, Deming Jin

**Affiliations:** 1 MOA Key Laboratory of Crop Ecophysiology and Farming System in the Middle Reaches of the Yangtze River, College of Plant Science and Technology, Huazhong Agricultural University, Wuhan, China; 2 Departments of Crop and Soil Science, University of Port Harcourt, Port Harcourt, Nigeria; New Mexico State University, UNITED STATES

## Abstract

The introduction of closely related species genomic fragments is an effective way to enrich genetic diversity and creates new germplasms in crops. Here, we studied the genetic diversity of an introgression line (IL) population composed of 106 ILs derived from an interspecific tetra cross between *O*. *glaberrima* and *O*. *sativa* (RAM3/Jin23B//Jin23B///YuetaiB). The proportion of *O*. *glaberrima* genome (PGG) in the ILs ranged from 0.3% to 36.7%, with an average value of 12.32% which is close to the theoretically expected proportion. A total of 250 polymorphic alleles were amplified by 21 AFLP primer combinations with an average of 12 alleles per primer. Population structure analysis revealed that the IL population can be divided into four genetically distinct subpopulations. Both principal component analysis and neighbor-joining tree analysis showed that ILs with a higher PGG displayed greater genetic diversity. Canonical discriminant analysis identified six phenotypic traits (plant height, yield per plant, filled grain percentage, panicle length, panicle number and days to flowering) as the main discriminatory traits among the ILs and between the subpopulations and showed significant phenotypic distances between subpopulations. The effects of PGG on phenotypic traits in the ILs were estimated using a linear admixed model, which showed a significant positive effect on grain yield per plant (0.286±0.117), plant height (0.418 ± 0.132), panicle length (0.663 ± 0.107), and spikelet number per panicle (0.339 ± 0.128), and a significant negative effect on filled grain percentage (-0.267 ± 0.123) and days to flowering (-0.324 ± 0.075). We found that an intermediate range (10% − 20%) of PGG was more effective for producing ILs with favorable integrated agronomic traits. Our results confirm that construction of IL population carrying *O*. *glaberrima* genomic fragments could be an effective approach to increase the genetic diversity of *O*. *sativa* genome and an appropriate level of PGG could facilitate pyramiding more favorable genes for developing more adaptive and productive rice.

## Introduction

The cultivated rice species *Oryza sativa* and *Oryza glaberrima* were independently domesticated in Southern Asia and Western Africa, respectively, from different wild species progenitors [1]. The higher yielding *O*. *sativa* is now cultivated globally, whereas *O*. *glaberrima* is largely restricted to West Africa. However, *O*. *glaberrima* varieties offer distinct advantages such as weed competitiveness, high light use efficiency, drought tolerance, and pest and disease resistance [2, 3, 4, 5, 6, 7, 8]. *O*. *glaberrima* has the ability to grow in a wide range of challenging ecosystems such as rain-fed hilly areas, deep-water conditions, and in coastal mangrove areas [9, 10]. Thus, this species could provide valuable germplasm resources for improving the environmental adaptation of Asian rice varieties.

The introduction of genes from evolutionarily distant germplasms into the *O*. *sativa* genome is a promising approach for breeding new inbred rice varieties. Introgression lines (IL) and chromosome segment substitution lines (CSSL) have been developed for the genetic improvement of rice via transfer of favorable traits from *O*. *glaberrima* into *O*. *sativa* [11, 12, 13, 14, 15]. The New Rice for Africa (NERICA) varieties were derived from interspecific crosses of *O*. *sativa* and *O*. *glaberrima* at the African Rice Center. The NERICA varieties are high-yielding, drought and pest resistant, and are adapted to the local conditions of West Africa [10, 16]. Furthermore, the introgression of *O*. *glaberrima* genomic fragments into drought susceptible *O*. *sativa* backgrounds can enhance grain yield under drought stress [17]. Quantitative trait loci (QTLs) for plant height, tiller number, panicle number, sterility percentage, 1000-grain weight, and grain yield have been investigated in chromosome segment substitution lines carrying *O*. *glaberrima* genes [18]. Therefore, it is possible to combine the high yield potential and low yield loss due to stress into ILs carrying African rice genes [19, 20].

Recently, further utilization of ILs carrying African genes is considered a promising approach in breeding hybrid rice varieties. A new type of CMS rice with African rice (*O*. *glaberrima)* cytoplasm has been developed to increase the cytoplasmic genetic diversity of three-line hybrid rice; a new dominant restorer gene was identified from *O*. *glaberrima* in introgression restorer lines [21]. The development of partial inter-specific hybrid rice by crossing sterility lines of *O*. *sativa* and introgression restorer lines carrying *O*. *glaberrima* genes has been reported to result in a positive interspecific heterosis for grain yield [22].

Information of population structure and genetic diversity of the germplasm is very important for both genetic researches and breeding projects [23, 24]. Many researches have been reported on genetic diversity of the IL population which derived from the interspecific cross of different crops, such as rice [25, 26, 27], bean [28], barely [29], and cotton [24]. However, previous researches on genetic diversity in rice were focused on IL populations that derived from crosses between various wild rice (*Oryza rufipogen* etc.) and Asian rice (*Oryza sativ*a) [25, 26, 27]. Knowledge on genetic diversity and population structure of IL population that derived from the interspecific cross between *Oryza glaberrima* and *Oryza sativa* is limited. The effect of *O*. *glaberrima* introgression segments on the phenotypic traits of the IL population has not been reported.

A variety of molecular markers can be used to evaluate the genetic diversity of populations, such as simple sequence repeats (SSRs) and amplified fragment length polymorphisms (AFLPs). SSR markers are codominant, generally locus specific, and randomly dispersed throughout the plant genome but need completed genome sequence information to determine their exactly location on the genome [30, 31]. Although the genome sequence information is readily available for *Oryza sativa*, it is still inadequate for *O*. *glaberrima* [32]. AFLPs, however, combine the strengths of restriction fragment length polymorphism (RFLP) and rapid amplified DNA (RAPD) markers. They can reveal abundant polymorphic information using a small number of primer combinations [33] independent of prior knowledge of the genome sequence [34]. Therefore, AFLP has its own advantages as compared to SSR for population genetics study. Usually only a few primer combinations are needed for studying the genetic diversity. For example, genetic structure and diversity analysis were revealed by four AFLP selective primer combinations on different *Echinochloa* spp. [35]. Sixteen AFLP primer combinations were used for assessment of the genetic diversity of *Calotropis procera* in the West Africa region [36]. Eight AFLP primer combinations were used to analyze genetic structure of *Oryza glumaepatula* wild rice populations in Costa Rica [37]. In this study, we selected twenty one AFLP primer combinations for evaluating genetic diversity of the IL population.

The objectives of this investigation were to (1) estimate the proportion of *O*. *glaberrima* genomic fragments (PGGs) in the ILs; (2) to analyze the genetic diversity and phenotypic variation of ILs with different PGGs; (3) to evaluate the impact of PGG on phenotypic variation of agronomic traits of the ILs.

## Materials and Methods

### Plant materials

A set of 131 ILs were derived from an interspecific tetra cross RAM3/Jin23B//Jin23B///YuetaiB. Data of 106 ILs was obtained. RAM3 is a genetically representative accession of *O*. *glaberrima* [38], while Jin23B and YuetaiB are *O*. *sativa* varieties and maintainer lines for indica CMS lines widely used for hybrid rice production in China [22].

### Phenotypic evaluation

The ILs were grown at Huazhong Agricultural University (30°28´17˝N, 114°21´56˝W), Wuhan, Hubei province of China, in the summer season of 2012. A randomized complete block design with three replications was used and each plot contained 24 plants in two rows with 15 × 20 cm spacing. Eight phenotypic traits were evaluated: days to flowering (DF); plant height (PH); panicle length (PL); panicle number per plant (PN); spikelet number per panicle (SN); filled grain number per panicle (FGN); thousand grain weight (TGW); and yield per plant (YP). FGP was calculated as follows:

$$\text{FGP}\left(\text{\%}\right)=\frac{FGN}{SN}\times 100$$

where FGN and SN were obtained from the three representative plants.

### DNA extraction and AFLP analysis

Three grams of fresh leaves were ground in liquid nitrogen using a pestle and mortar. Total genomic DNA extraction was performed using the cetyltrimethy lammonium bromide method (CTAB) as described by Adedze et al. (2015) with minor modifications [22]. The DNA was dissolved in 200 μl sterile deionized water. The quality of the isolated DNA was checked on the 1% agarose gel and the quantity was determined using an ND-1000 spectrophotometer (NanoDrop Technologies, USA).

AFLPs were detected using a method similar to that described by Vos et al. [39]. The AFLP products were separated on a denaturing polyacrylamide gel and visualized by silver staining. The electrophoretic bands were scored as 1 (present) or 0 (absent) to form a raw data matrix for further analysis.

### Data analysis

#### Genetic diversity and cluster analyses

AFLPs were used to assess genetic diversity by determining the number of alleles, the number of polymorphic bands, the proportion of polymorphism (%), Nei’s genetic diversity index, and the Shannon diversity information index. POPGENE 3.2 software was used to calculate Nei’s genetic diversity index and the Shannon diversity information index. Two hundred and fifty five AFLP alleles which showed dominance (present) in all three parents but recessiveness (absent) in some of the ILs were selected to estimate the frequency of variable loci (FVL) as follows:

$$\text{FVL}\left(\text{\%}\right)=\frac{RL}{RL+DL}\times 100$$

where RL is the number of recessive loci and DL is the number of dominant loci in the target IL.

The STRUCTURE software program was used to infer the structure of the IL population [40]. Ten independent runs were performed with the number of subpopulations (*K*) ranging from 1 to 10 with the admixture model, a burn-in period length of 5000, a run length of 50,000, and 10 replications for each *K* value. Both LnP(D) and Evannos’s Δ*K* methods were used to estimate the number of subpopulations. The run of the estimated numbers of subpopulations showing the maximum likelihood was used to assign genotypes with membership probability ≥ 0.65 to subpopulations. Genotypes with membership probability ≤ 0.65 were assigned to an admixed group. A principal component analysis (PCA) was carried out using the Eigen program of NTSYS-pc 2.1e based on the DIST (average taxonomic distance) coefficient. Genetic and phenotypic differentiations in the IL population were estimated by constructing a neighbor-joining (NJ) tree in MEGA 5.0. The SAS software (SAS Institute, Cary, NC, USA) was used for statistical analyses of the phenotypic data. The phenotypic differences among ILs and subpopulations (subpopulations generated by structure analysis) were assessed via the canonical discriminant analysis using the CANDISC procedure [41]. Canonical discriminant analysis is a dimension-reduction technique related to principal component and canonical correlation analyses. It derives canonical discriminant functions that have the highest possible multiple correlation with groups and summarizes among-class variation in a similar way that principal component analysis summarizes total variation [42].

#### Estimation of PGG

The proportion of *O*. *glaberrima* fragments in the genome (PGG) of each IL was estimated with a Bayesian method according to the admixture model described by Pritchard et al. [43] and Gouy et al. [44]. PGG was obtained using STRUCTURE 2.3.4 as a Q-vector, according to 10 simulations at *K* = 2, referring to the genetic sources of *O*. *glaberrima* and *O*. *sativa*; a burn-in period length of 10,000 and a run length of 100,000 were performed. The probability of membership for the cluster including RAM3, from the run with lowest log likelihood value (-4593.1), was used to estimate the PGG of each IL. The plot of the STRUCTURE results was generated by the DISTRUCT program.

#### Estimation of PGG effects on phenotypic traits

The effect of PGG on phenotypic traits was assessed via the following mixed model:

$$\text{y}=\text{xa}+z_{1}b+z_{2}d+e$$

where y is the vector of phenotypic observations of a given trait, x is the vector of PGG in each IL, a is the fixed effect of PGG, b is the vector of genetic variation random effects within groups according to the pedigree ~N(0, σ_b_^2^I), d is the vector of random effects within different pedigrees ~N(0, σ_d_^2^I), I is the identity matrix, e is the vector of residual error of the model, and z_1_ and z_2_ are incidence matrices. A likelihood ratio test was performed to test for the fixed effect of the proportion of the genome arising from *O*. *glaberrima*.

To avoid systemic errors due to different dimensions, a standardizing treatment was performed by a zero-mean normalization before the data were used for the mixed model. All these models were computed using SPSS 19 software (SPSS Inc., Chicago, IL, USA).

## Results

### AFLP diversity and PGG in the genomes of ILs

Twenty-one primer combinations showing rich molecular diversity were selected to produce the AFLP profiles for the 106 ILs evaluated in this study. A total of 533 alleles were identified. The number of bands produced by the primer combinations ranged from 18 (Eatc/Mcag) to 39 (Ega/Mcaa). The number of polymorphic bands per primer combination varied from 5 (Ega/Mcta) to 24 (Eaag/Mtag), with an average of 12 bands per combination (Table 1). The proportion of polymorphisms identified by each primer combination ranged from 20.83% (Ega/Mcta) to 94.44% (Eatc/Mcag), with an average of 46.9%. Nei’s genetic diversity index averaged 0.2198 (range 0.1274 to 0.3204) and the Shannon diversity information index averaged 0.3470 (range 0.2159 to 0.4886) (Table 1).

**Table 1:** Polymorphism and molecular diversity analysis of AFLP markers.

Primer combination	Observed number of alleles	Number of polymorphic bands	Polymorphic percentage(%)	Shannon information index	Nei’s diversity index
**Eagg/Mctt**	25	18	72.00	0.2206	0.3621
**Ega/Mcta**	24	5	20.83	0.1274	0.2289
**Ega/Mcaa**	39	9	23.08	0.1963	0.3043
**Ega/Mtag**	28	13	46.43	0.2410	0.3718
**Eaaa/Mcct**	26	11	42.31	0.3249	0.4886
**Eata/Maag**	21	10	47.62	0.2547	0.3903
**Eagg/Mcat**	27	9	33.33	0.1537	0.2652
**Eaag/Mcag**	25	8	32.00	0.2386	0.3755
**Eagt/Mcac**	33	15	45.45	0.1669	0.2714
**Eaag/Mtag**	26	24	92.31	0.2811	0.4308
**Eaaa/Mctt**	20	9	45.00	0.1664	0.2850
**Eaca/Mcca**	18	15	83.33	0.1522	0.2661
**Eatc/Mcag**	18	17	94.44	0.2568	0.4130
**Eacc/Mcgt**	22	7	31.82	0.2180	0.3622
**Eaac/Mctc**	30	12	40.00	0.2255	0.3554
**Ega/Mctg**	24	9	37.50	0.1206	0.2159
**Eaaa/Mcta**	30	19	63.33	0.2852	0.4458
**Eata/Mctt**	24	11	45.83	0.3204	0.3889
**Ega/Maag**	30	13	43.33	0.2074	0.3459
**Eacc/Mcaa**	21	10	47.62	0.3031	0.4487
**Eagc/Mcat**	22	6	27.27	0.1541	0.2716
**Mean**	25.38	11.90	48.33	0.2198	0.3470
**Total**	533	250	-	-	-

**Fig 1:**
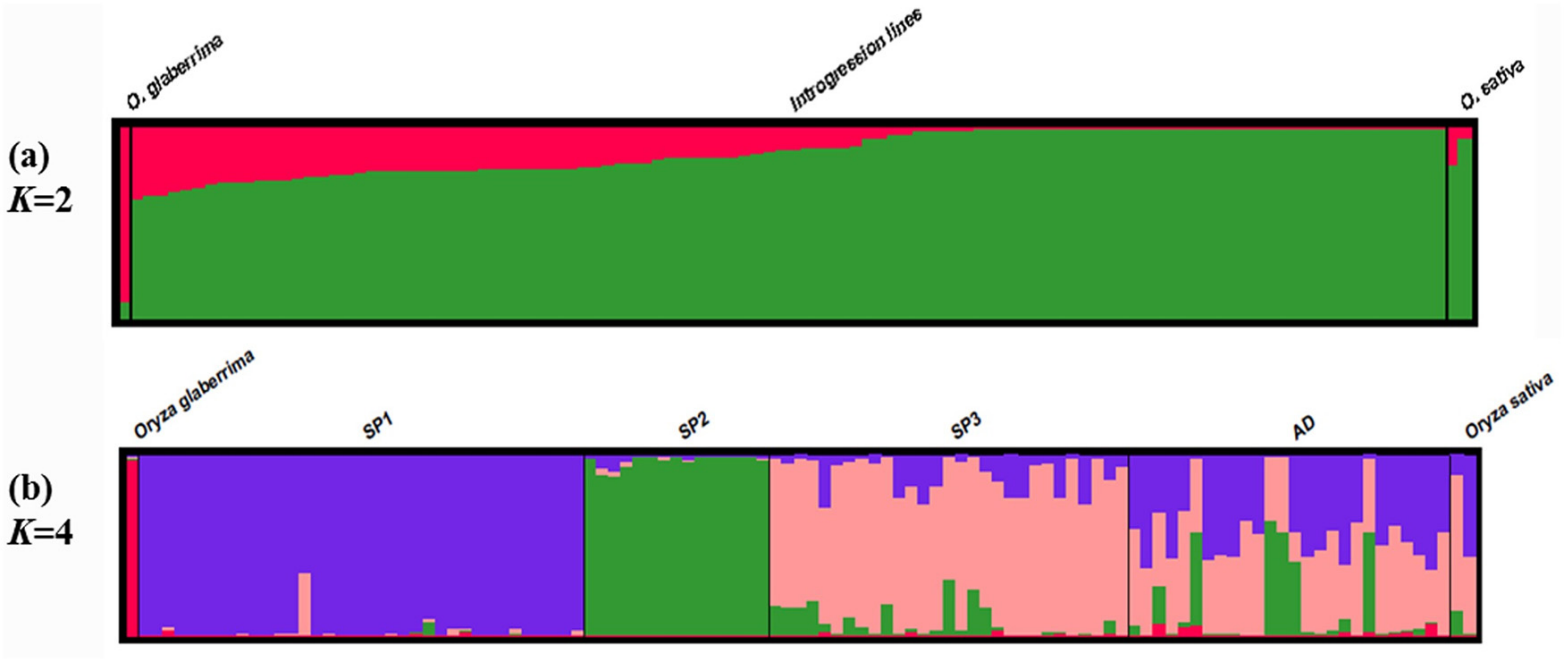
Estimated population structures of the IL population with *K* = 2 (a) and *K* = 4 (b). The ILs were sorted according to the Q vector of cluster 1(a) and the max Q vectors in each subpopulation (b).

**Fig 2:**
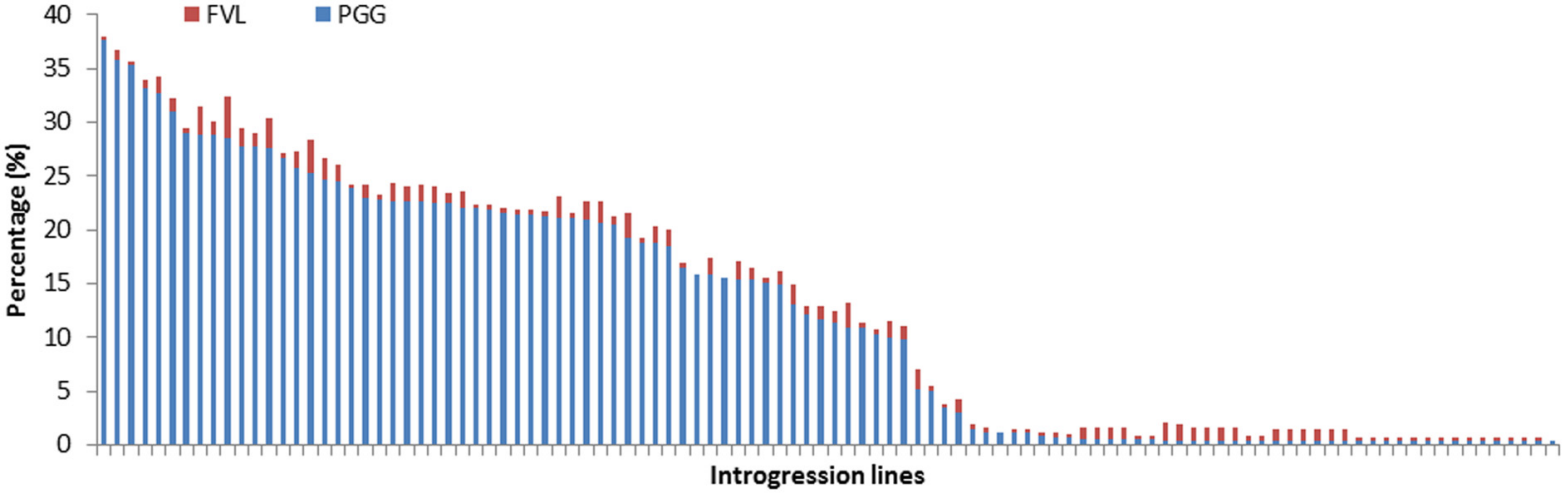
PGG and frequency of variable loci (FVL) in the ILs.

STRUCTURE version 2.3.4 was used to compute a Q-vector containing the estimated proportion of *O*. *glaberrima* fragments in the genome for every IL and identify the population structure (Fig 1). As the ILs used in this study came from a multiple cross between *O*. *sativa* and *O*. *glaberrima*, the *K* number of population was set at two to estimate the PGG of each IL (Fig 1a). The analysis indicated that PGG ranged from 0.3% to 37.6%, with an average of 12.32%; this average is close to the expected theoretical ratio of 1/8 (12.5%) in the interspecific tetra cross, indicating the ILs essentially represented a random sample population. The frequency of variable loci in ILs ranged from 0% to 3.92%, with an average of 0.98% (Fig 2).

### Population structure and genetic diversity

**Fig 3:**
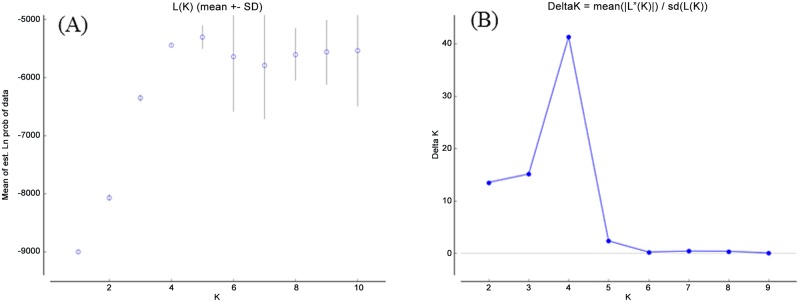
The L(*K*) and Δ*K* values with different *K* value calculated by STRUCTURE Harvester. A: *K* versus mean in likelihood. B: Magnitude of Δ*K* as a function of *K*. Note the steady increase in likelihood from *K* = 1 to *K* = 10, and the relatively highest value of Δ*K*, with peaks at *K* = 4.

The optimum population structure inferred by STRUCTURE was four clusters according to both LnP(D) and Evannos’s Δ*K* methods (*K* = 4; Fig 3). With the membership probability ≥ 0.65, three subpopulations were identified in the IL population and denoted as SP1, SP2 and SP3. These subpopulations contained 36, 15, and 29 ILs, respectively. Twenty-six ILs were retained in the admixed group (AD) (Fig 1b). Net nucleotide distances indicated that the highest degree of differentiation within subpopulations was present between SP1 and SP2 (0.0781), and the lowest between SP1 and SP3 (0.0546). The average genetic distances within the three subpopulations were 0.0136, 0.0228, and 0.1254, respectively.

A principal component analysis (PCA) based on marker genotypes was performed to determine the consistency of the differentiation of the populations defined by the cluster analysis (S1 Fig). The analysis showed that the first three principal components explained 25.10%, 12.35% and 7.96% of the molecular variance. These components therefore accounted for a total of 45.41%. The result of PCA showed that most ILs (47/48) with lower PGG (< 10%) were clustered into 2 groups, Cluster I (37), Cluster II (10), while Cluster III contained 12 ILs with intermediate PGG (10% − 30%). The remaining 46 ILs with intermediate or higher PGG (10% − 40%) were distributed discretely, indicating higher PGG might lead to greater variation between the genotypes.

**Fig 4:**
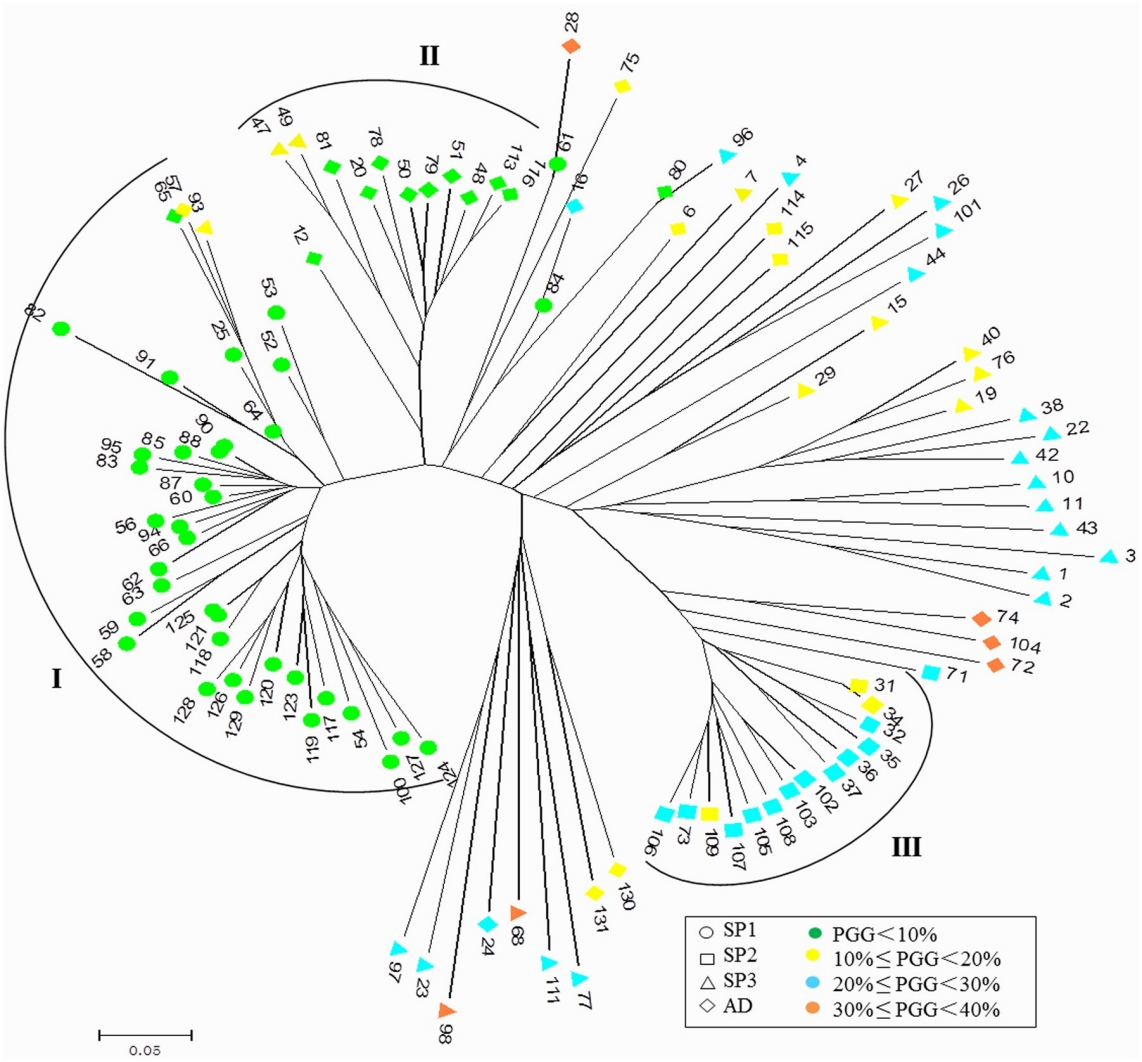
Unrooted neighbor-joining phylogenetic trees based on AFLP markers in the ILs. There were 35 and 10 ILs with lower PGG (< 10%) in Cluster I (37) and Cluster II (12), respectively. while Cluster III contained 14 ILs with intermediate PGG (10% − 30%). The remaining 44 ILs with intermediate or higher PGG were scattered in different clades.

**Fig 5:**
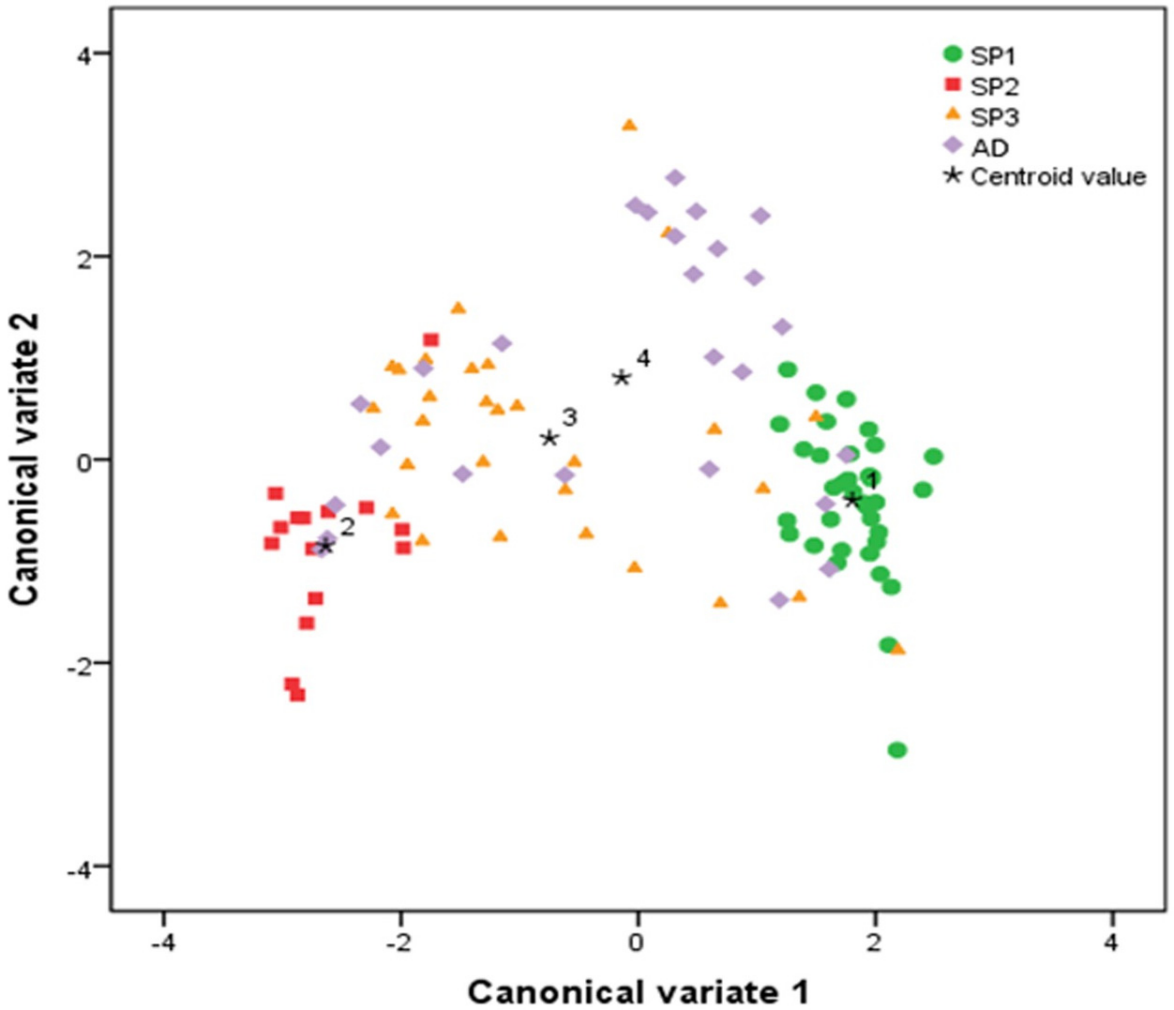
Scatterplot of the ILs belonging to different subpopulations based on the first two canonical discriminant functions.

An NJ tree based on the AFLP markers showed clusters similar to that of the PCA and STRUCTURE analysis (Fig 4). The genetic distance between ILs ranged from 0.00 to 0.65 with an average value of 0.1893. At a coefficient value of 0.15, 65 ILs (61.32%) were assigned into three clusters and 41 ILs (38.68%) were scattered into different clades. The three clusters, termed Cluster I, II, and III, included 37, 12, and 14 ILs, respectively. A combined analysis with the PGG levels of the ILs in the clusters showed that most ILs (45/48) with lower PGG (< 10%) concentrated in Clusters I and II. while Cluster III contained 14 ILs with intermediate PGG (10% − 30%). The remaining 44 ILs with intermediate or higher PGG (10% − 40%) were scattered in different clades at a coefficient value of 0.15, and showed greater genetic distances than that between ILs in Clusters I and II (Fig 5). These results indicate that genetic variation may increase in ILs that carried higher proportions of *O*. *glaberrima* fragments.

### Phenotypic variation of ILs

Significant variation for eight agronomic traits was observed among the ILs (Table 2), indicating diverse morphologic performance in this IL population. Plant height ranged from 98 cm to 143.33 cm, with an average of 116.86 cm. Yield per plant ranged from 17.88 g to 55.60 g, with an average of 34.90 g, while thousand grain weight ranged from 17.78 g to 31.63 g, with an average of 22.89 g. Spikelet number ranged from 79.40 to 174.92. The filled grain percentage ranged from 50% to 91%. Effective tiller number varied from 8.33 to 18.00. Panicle length varied from 18.86 cm to 29.67 cm; and days to flowering ranged from 67 to 104 days.

**Table 2:** Agronomic traits variation in ILs.

Trait	Minimum	Maximum	Mean	SD	P
**PH (cm)**	98.00	143.33	116.86	7.74	< 0.01
**YP (g)**	17.88	55.60	34.90	6.88	< 0.01
**TGW (g)**	17.78	31.63	22.89	2.00	< 0.01
**SN (no/plant)**	79.40	174.92	128.22	19.69	< 0.01
**FGP (%)**	50.00	91.00	75.85	10.21	< 0.01
**PL (cm)**	18.86	29.67	24.96	2.34	< 0.01
**PN (no/plant)**	8.33	18.00	11.96	1.86	< 0.01
**DF(d)**	67.00	104.00	88.20	12.67	< 0.01

PH = plant height; YP = yield per plant; TGW = thousand grain weight; SN = spikelet number per panicle; FGP = filled grain percentage; PL = panicle length; PN = panicle number per plant; DF = days to flowering.

An NJ tree (S2 Fig) was performed for the agronomic traits of ILs using DIST distance coefficients, which varied from 0.139 to 3.165 with an average of 1.326. Most ILs (43/48) with lower PGG (< 10%) were assigned into two clusters on the phenotypic NJ tree. There were two smaller clusters. One contained mainly ILs (9/10) with PGGs in the range 0% −20%, and another contained 10 ILs with PGGs in the range 10% − 30%. The remaining 38 ILs with PGGs (10% − 40%) were dispersed to different branches. The phenotypic NJ tree showed that ILs with low PGG (< 10%) were more closely distributed, indicating these lines have lower phenotypic variation.

The canonical coefficients showing the contribution of each measured trait to the total variation are presented in Table 3. The canonical correlation measures the strength of the overall relationships between the linear composites of predictor (canonical discriminant variate) and criterion (cultivars) sets of variables [41]. The significant (p < 0.001) canonical correlations between the ILs and the first canonical variate (r_c1_ = 0.836) and between the ILs and the second canonical variate (r_c2_ = 0.504) indicate that this canonical variates can explain the phenotypic differentiation of this IL population. The first canonical discriminant function (canonical variate 1) explained 84.3% of the variance and was dominated by large coefficients from the plant phenotypic traits—yield per plant, panicle number per plant and days to flowering. Canonical variate 2 accounted for 12.3% of the variation and was dominated by large coefficients from this traits—plant height, filled grain percentage, panicle length and panicle number per plant. The two canonical variates together accounted for 96.5% of the variation among ILs.

**Table 3:** The canonical loadings of the measured traits on the first two canonical variables of the ILs.

Phenotypic trait	Canonical variate	
	1	2
**Plant height (PH)**	-0.172	0.568
**Yield per plant (YP)**	0.356	-0.058
**Thousand grain weight (TGW)**	-0.105	-0.105
**Spikelet number per panicle (SN)**	0.027	0.002
**Filled grain percentage (FGP)**	-0.052	0.471
**Panicle length (PL)**	0.117	0.407
**Panicle number per plant (PN)**	-0.328	0.530
**Days to flowering (DF)**	-0.829	0.143
**Canonical correlation**	0.837	0.504
**p level of significance**	<0.0001	0.0005
**variance accounted for**	0.843	0.123

The centroid values of the first two canonical discriminant functions for the accessions were plotted as the *x* and *y* axis to visualize the subpopulation clusters (Fig 5). The extent of separation between the subpopulation clusters was measured by *D*^2^. The greatest phenotypic distance was observed between SP1 and SP2 (*D*^2^ = 19.96) followed by SP2 and AD (*D*^2^ = 8.96), SP1 and SP3 (*D*^2^ = 7.12), SP1 and AD (D^2^ = 5.32), SP2 and SP3 (*D*^2^ = 5.20), and SP3 and AD (*D*^2^ = 1.31). All pairwise distances between the subpopulation clusters were significant.

Agronomic performance in ILs was further analyzed with respect to population structure based on AFLP markers. Significantly differences were found between the means of subpopulations for all the traits (S1 Table). Plant height showed the highest mean (121.00 cm) in admixture subpopulation (AD) and the lowest (110.73 cm) in SP2. Spikelet number had the highest average in the SP2 (145.27) and the lowest in the SP1 (112.77). The longest panicles were found in the SP2 (27.26 cm) while the shortest panicles were in SP1 (22.79 cm). The latest flowering plants were in the SP1 (100.78 d) while the earliest flowering ILs were in SP2 (70.33 d). The AD had the highest filled grain percentage (81%) while the SP2 had the lowest filled grain percentage (67%). The highest grain yield per plant was in the AD (38.67 g) with the mean PGG of 12% while the lowest grain yield per plant showed in SP1 (30.18 g) with the mean PGG of 1%.

### Comparison of genetic distances between ILs based on molecular markers and phenotypic variation

**Fig 6:**
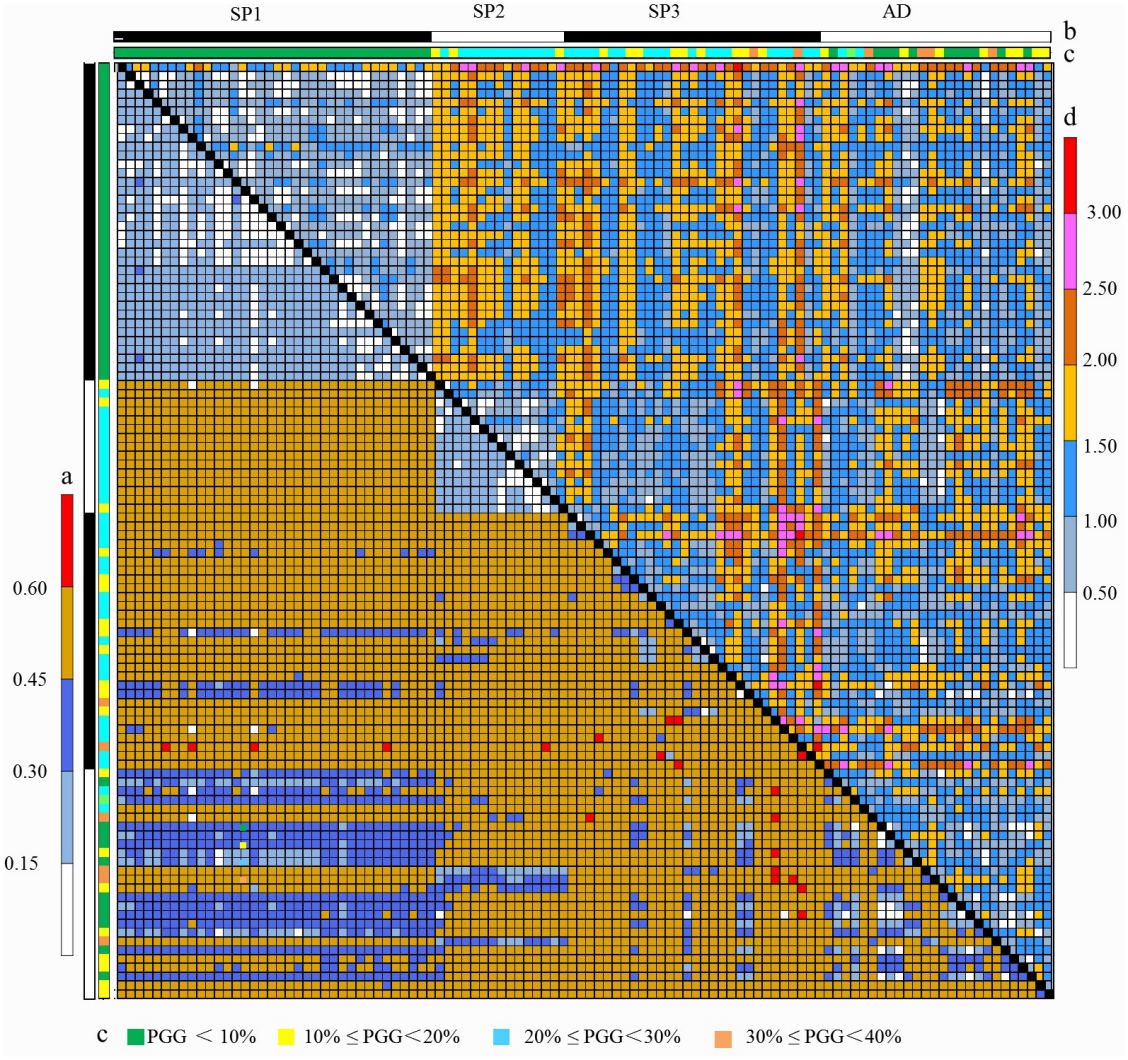
Comparison of two matrixes of pairwise DIST distances between ILs based on AFLP markers and phenotypic traits. Lower left (a): Matrix of pairwise DIST distances between ILs based on AFLP markers; (b): The structure of the IL population; Upper right (c): The PGG in ILs; (d): Matrix of pairwise DIST distances between ILs based on phenotypic traits.

Comparison of the genotypic and phenotypic DIST distance matrixes in the ILs revealed both similarities and differences (Fig 6). Genetic distances between ILs based on phenotypic traits (0.139 − 3.165) were generally higher than those based on molecular markers (< 0.65). However, genetic distances between ILs from the two sets of data showed similar trends.

Genotypic and phenotypic DIST distances varied between ILs within different subpopulations. For ILs of SP1 and SP2, genotypic and phenotypic distances were relatively low, < 0.30 and < 1.50, respectively, while for most ILs of SP3, they were relatively high, > 0.45 and 1.00 − 3.50, respectively.

Larger genetic distances were present in ILs from different subpopulations. The average genotypic and phenotypic distances between SP1 and SP2 were 0.459 and 1.621, respectively. The average genotypic and phenotypic distances between ILs from SP1 and SP3 were 0.480 and 2.732 respectively; those between SP2 and SP3 were 0.490 and 1.385, respectively.

Exceptionally high genotypic and phenotypic genetic distances were found between ILs with high PGGs (Fig 6). The top 10 highest phenotypic distances (≥ 2.85) were detected between 10 pairs of ILs with an average PGG of 22.42%; the top 10highest genotypic distances (≥ 0.61) occurred between ILs with an average PGG value of 23.80%. Both values were much higher than the overall mean of 12.39% in the ILs.

### Effects of *Oryza glaberrima* genomic fragments on phenotypic traits in ILs

The relationship between the PGG in ILs and eight phenotypic traits was examined using Pearson’s correlation analysis (Table 4). Significant positive correlations were identified for yield per plant (*r* = 0.275**), spikelet number per panicle (*r* = 0.415**), panicle length (*r* = 0.583**), and negative correlations were found for thousand grain weight (*r* = -0.264**), filled grain percentage (*r* = -0.503**), days to flowering (*r* = -0.707**). No significant correlation was found for plant height or effective tiller number.

**Table 4:** Pairwise Pearson’s correlations between PGG or FVL and phenotypic traits in ILs.

	Z(YPP)^a^	Z(PH)	Z(TGW)	Z(SN)	Z(FGP)	Z(PL)	Z(EPN)	Z(DF)
**Z(PGG)**	0.275**	-0.186	-0.264**	0.415**	-0.503**	0.583**	0.153	-0.707**
**Z(FVL)**	0.074	-0.342**	-0.213*	0.173	-0.412**	0.301**	0.124	-0.480**

PH = plant height; YP = yield per plant; TGW = thousand grain weight; SN = spikelet number per panicle; FGP = filled grain percentage; PL = panicle length; PN = panicle number per plant; DF = days to flowering; PGG = the proportion of *O*. *glaberrima* genome; FVL = the frequency of variable loci.

^a^Correlation coefficients were calculated with zero-mean normalization of each tested variate.

* Significance level < 0.05.

** Significance level < 0.01.

The effect of different levels of PGG on phenotypic traits was analyzed by the mixed model using the frequency of variable loci (FVL) as the co-variate (Table 5). PGG had a significant effect (P < 0.01) on plant height (0.418 ± 0.132), panicle length (0.663 ± 0.107), and days to flowering (-0.324 ± 0.075); the effect on yield per plant (0.286 ± 0.117) and spikelet number per panicle (0.339 ± 0.129) showed a lower level of significance (P < 0.05). PGG had a significant negative effect (P < 0.05) on filled grain percentage (-0.2679 ± 0.123).

**Table 5:** Effect and significance of PGG on phenotypic traits.

	Effect of Z(PGG)^a^	Effect of PGG^b^		
Traits	Estimated values	Units	Estimated values	P-value
**PH**	0.418 ± 0.132	cm	3.235 ± 1.018	0.002
**YP**	0.286 ± 0.117	g/plant	1.968 ± 0.808	0.018
**TGW**	0.070 ± 0.143	g/1000 grains	0.139 ± 0.285	0.626
**SN**	0.339 ± 0.128	No./panicle	6.670 ± 2.517	0.011
**FGP**	-0.267 ± 0.123	%	-2.338 ± 1.072	0.032
**PL**	0.663 ± 0.107	cm	1.551 ± 0.249	< 0.0005
**EPN**	0.282 ± 0.147	No./plant	0.524 ± 0.274	0.059
**DF**	-0.324 ± 0.075	d	-4.107 ± 0.952	< 0.0005

PH = plant height; YP = yield per plant; TGW = thousand grain weight; SN = spikelet number per panicle; FGP = filled grain percentage; PL = panicle length; PN = panicle number per plant; DF = days to flowering; PGG = the proportion of O. glaberrima genome; FVL = the frequency of variable loci.

^a^Estimated effects for Z scores of the traits;

^b^Estimated effects for the observed trait values.

**Fig 7:**
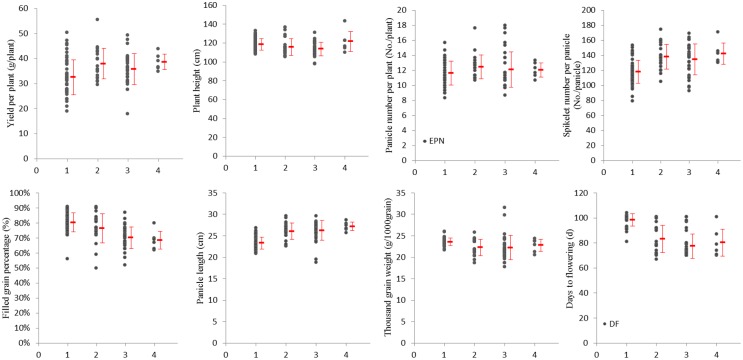
Phenotypic traits in ILs with different PGGs. 1: PGG < 10%, 2: 10% ≤ PGG < 20%, 3: 20% ≤ PGG < 30%, 4: 30% ≤ PGG < 40%.

To visualize the PGG effects to phenotypic traits, the performance of 8 phenotypic traits in four groups of ILs with different amounts of PGG (< 10%, 10% − 20%, 20% − 30%, 30% − 40%) are presented in Fig 7. Considerable phenotypic variation was present for all traits in all four groups of ILs; moreover, the extent of variation differed among the IL groups. Yield per plant, spikelet number per panicle, panicle number per plant, and panicle length tended to increase in ILs with higher PGGs; the reverse tendency was observed for plant height, thousand grain weight and filled grain percentage. The ILs of second PGG groups (10% − 20%) tended to show higher advantageous positive variation, such as bigger panicles and lower disadvantageous negative variation, such as reduced fertility. This suggests that the intermediate range (10% − 20%) of introgression of *O*. *glaberrima* genomic fragments might be more favorable for improving agronomic traits.

## Discussion

### Estimation of PGG in the ILs

There are several methods to estimate the introgression level of donor parent genome in ILs. A simple method is to calculate the frequency of alleles derived from donor parent in each IL [22]. Recently, Bayesian method according to the admixture model described by Pritchard et al. [43] was used to estimate the percentage of introgression fragments in ILs. In this study, we compared the PGG of each IL estimated by two different methods and found that the Bayesian method is more suitable for estimating the introgression level of *O*. *glaberrima* genomic fragments in the ILs. The average PGG (12.32%) of the ILs estimated by Bayesian method is close to the theoretically expected introgression level of 12.5%, while the frequency of introgression alleles showed an average of 5.53%. Although PGG values resulted from the two methods are different, they are significant correlated (R^2^ = 0.633, P < 0.05) (Fig 2, S3 Fig). Additionally, PGG based on Bayesian method showed more significant effects on phenotypic traits in the ILs than that estimated by frequency of introgression alleles. It suggests that PGG based on Bayesian method is more effective for analyzing the integrated effects of introgression fragments on phenotypic traits in the *O*. *sativa* background.

### Genetic and phenotypic diversity in the IL population

ILs incorporating African rice genomic fragments have been developed and studied for different purposes, such as identification and screening on yield related traits of ILs under biotic and abiotic stress, etc. [18, 19, 45]. However, little is known about the genetic diversity and phenotypic variation of IL populations in relation to the level of introgression of *O*. *glaberrima* genomic fragments into the *O*. *sativa* genome.

In this study, four subpopulations were detected with STRUCTURE in a population consists of 106 ILs based on 250 polymorphic AFLP sites, and greater genetic variation was found in subpopulations containing ILs with higher GGP. PCA and NJ phylogenetic tree analysis were constructed based on molecular markers to analyze the genetic differentiation, both results showed the ILs with lower PGGs (< 10%) were mostly assigned into two clusters while the ILs with high PGGs (10% − 40%) were distributed discretely, indicating higher PGG might lead to greater variation between the genotypes. There was a significant correlation between PGG level and the proportion of variable sites, with a correlation coefficient of 0.725 (P < 0.01). These results indicate that the introduction of *O*. *glaberrima* fragments into *O*. *sativa* genome background is an effective way to increase the genetic diversity of rice.

It is important to exploit the genetic diversity found in rice genetic resources which can be classified according to the phenotypic variability of agronomic traits [46]. In this study, significant phenotypic variation of agronomic traits was observed between individual rice genotypes and among the genetically defined subpopulations. The first two canonical variables in CDA accounted for 96.5% of the diversity that exists among the 106 ILs and six of the eight agronomic traits contributed largely to the diversity captured by the first two canonical variates. These six discriminatory traits included three yield traits (panicle number per plant, fertile grain percentage and yield per plant), two morphological traits (panicle length and plant height), and one photoperiod response character (days to flowering). These canonical variate functions indicate that more prominent variations of the genetic compositions of subpopulations exist in these six morphological, physiological and yield traits.

The yield related traits were found to be significantly different between pairs of subpopulations carry different PGGs as revealed by the t tests (S1 Table). The ILs in SP1 which has lower mean PGG (1%) produced the lower average yield per plant (30.18 g) and had relatively more inferior traits, while the AD group with relatively higher mean PGG (12%) had highest average yield per plant (38.67 g) and more favorable traits. Although SP2 which has higher mean PGG (21%) produced biggest panicles, its yield per plant (36.37 g) was slightly lower than that of AD group due to its significantly lower filled grain percentage. This suggested that more favorable alleles could be accumulated to ILs with increased PGG; however, an optimal range of PGG could reduce the probability of the introgression of some inferior alleles such as interspecific sterility genes.

Genetic variation provides the potential for a species to adapt to adverse environmental changes [47]. Genetic diversity is also the foundation for heterosis, which is widely employed in breeding of high yielding hybrid rice varieties. Introgression restorer lines carrying *O*. *glaberrima* genes have been reported to develop partial interspecific hybrid rice express positive yield heterosis [22]. Our results of an appropriate level of *O*. *glaberrima* introgression effectively increase the genetic diversity would encourage and facilitate the use of interspecific hybridization for high yielding and more adaptive rice breeding programs.

### Impacts of *O*. *glaberrima* introgression level on agronomic traits

Grain yield has been reported to increase by more than 20% in the progeny of crosses between *O*. *sativa* and *O*. *glaberrima* [48]. In this study, we further estimated the level of introgression in the ILs and examined the effect of variation in PGG on various phenotypic traits (Fig 2, Table 5). Our analyses showed that increased PGG (ranging from 0% to 40%) had positive effects on yield per plant, plant height, panicle length, panicle number, and spikelet number per panicle, but negative effects on days to flowering, and filled grain percentage. Our analysis showed that increased PGG levels were associated with enhancement of some yield-related traits, such as larger panicle, but also an increase in some unfavorable traits, such as a lower filled grain percentage.

African rice has many favorable genes that could potentially be used to improve the agronomic traits of Asian rice, although it is inferior to Asian rice in yield related traits such as panicle size and plant type [49, 50]. Many QTLs for resistance genes have been identified in African rice, for example, for P-starvation tolerance, rice stripe necrosis virus resistance, and green rice leafhopper resistance [17, 51, 52]. The introgression of *O*. *glaberrima* genomic fragments into *O*. *sativa* allows the importation of genes controlling adaptive traits such as drought tolerance and biotic resistance [11], thus, these genomic fragments potentially could improve the stability of yield performance in ILs. In addition, the African rice also has genes that contribute to yield related traits. QTLs for plant height, tiller number, panicle length, and grain yield have also been identified [19, 20]. The panicle size of African rice is usually smaller than that of Asian rice mainly because the former does not have secondary branches in its panicle. However, in this study, all derived ILs and interspecific hybrids had panicles with secondary branches, and PGG level had a positive effect on spikelet number per panicle. Therefore, the accumulation and interaction of favorable genes introduced from African rice can improve adaptation and productivity of Asian rice both directly and indirectly.

Most *O*. *sativa* and *O*. *glaberrima* F_1_ hybrids are sterile due to the sexual barrier between the two species. Many genes responsible for this hybrid sterility have been identified, such as *S1*, *S3*, *S18*, *S19*, *S20*, *S21*, *S29(t)*, *S36(t)*, *S37(t)*, *S38(t)*, and *S39(t)* [53,54, 55, 56, 57]. Most of these genes are eliminated from ILs through selection. Nevertheless, we found here that PGG level had a significant negative effect on the filled grain percentage suggesting that some minor genes from *O*. *glaberrima* controlling interspecific sterility might persist in ILs with higher PGG levels.

In the present study, a low frequency of variable loci (FVL), which were distinct from both *O*. *glaberrima* and *O*. *sativa*, was detected in the ILs. We included the FVL in the mixed model to estimate the random effects of genetic variation as a co-variate to avoid its interference with our analysis of PGG effects. Significant correlations were found between FVL and PGG and between FVL and phenotypic traits, such as filled grain percentage and days to flowering (Table 4). This analysis suggested that the FVL was associated with PGG level and was an additional cause of genetic diversity and phenotypic variation in ILs.

While the introgression of *O*. *glaberrima* fragments is an effective way to broaden the genetic diversity and improve phenotypic traits, it is crucial to determine the optimal level of PGG for efficient development of elite ILs. Here, we found that a PGG level of 10% − 20% appeared to be most favorable; thus, the interspecific tetra cross with an average PGG of 12.5% was considered suitable for producing ILs for genetic improvement of yield related traits in *O*. *sativa*. The results of this study demonstrated that moderate introgression levels (10% − 20%) from the *O*. *glaberrima* genome can facilitate both the incorporation of beneficial genes and elimination of interspecific sterility genes to achieve high grain yield.

## Conclusion

An IL population derived from an interspecific cross between *Oryza glaberrima* and *Oryza sativa* was divided into four distinct subpopulations by STRUCTURE analysis. AFLP marker genotypes and morphological traits were analyzed to evaluate the PGG levels and its effects on genetic diversity and phenotypic variation in the ILs. An estimated population mean of PGG of 12.32% was found in the IL population, consistent with to the theoretical ratio of a random sample population. PCA and NJ tree analysis revealed greater genetic diversity and larger phenotypic variations in ILs with higher PGG levels. Canonical discriminant analysis showed great phenotypic distance between subpopulations, and six agronomic traits (plant height, yield per plant, filled grain percentage, panicle length, panicle number, and days to flowering.) were the main discriminatory characters among the ILs and between the subpopulations. PGG had positive effects on yield per plant, plant height, panicle length, spikelet number per panicle, and negative effects on filled grain percentage, and days to flowering. Additionally, we found an intermediate range (10% − 20%) of PGG was favorable for the incorporation of more beneficial genes and the elimination of harmful genes from *Oryza glaberrima*. Overall, our results could facilitate the further exploitation of *O*. *glaberrima* germplasm to breed more productive and adaptive inbred and hybrid rice varieties.

## Supporting Information

S1 Fig Three-dimensional scaling of ILs by principal component analysis (PCA) using AFLP markers. PC1, PC2 and PC3 refer to the first, second and third principal components, respectively. The numbers in parentheses refer to the proportion of the variance explained by the corresponding axes. Most ILs (47/48) with lower PGGs (< 10%) were clustered into two groups, Cluster I (37), Cluster II (10), while Cluster III contained 12 ILs with intermediate PGG (10% − 30%). The remaining 46 ILs with intermediate or higher PGG (10% − 40%) were distributed discretely.
(TIF)

S2 Fig Unrooted neighbor-joining tree based on phenotypic traits in ILs. Most ILs (43/48) with lower PGG (< 10%) were assigned into two clusters on the phenotypic NJ tree. There were two smaller clusters. One contained ILs (9/10) with PGGs in the range 0% − 20% and another contained 10 ILs with PGGs in the range 10% − 30%. The remaining 38 ILs with PGGs (10% − 40%) were dispersed to different branches.
(TIF)

S3 Fig The PGG in the ILs. (a) PGG in the ILs estimated by using introgression alleles. (b) Comparison of PGG estimated by Bayesian method vs. PGG estimated by using introgression alleles in ILs.
(TIF)

S1 Table Phenotypic and PGG variation between the 4 subpopulations. (DOCX)
