# Supplementary figures and images for: Genetic Diversity and Phenotypic Variation in an Introgression Line Population Derived from an Interspecific Cross between Oryza glaberrima and Oryza sativa
Source: PLoS One. 2016 Sep 7;11(9):e0161746. doi: 10.1371/journal.pone.0161746 (PMC5014448; doi:10.1371/journal.pone.0161746)

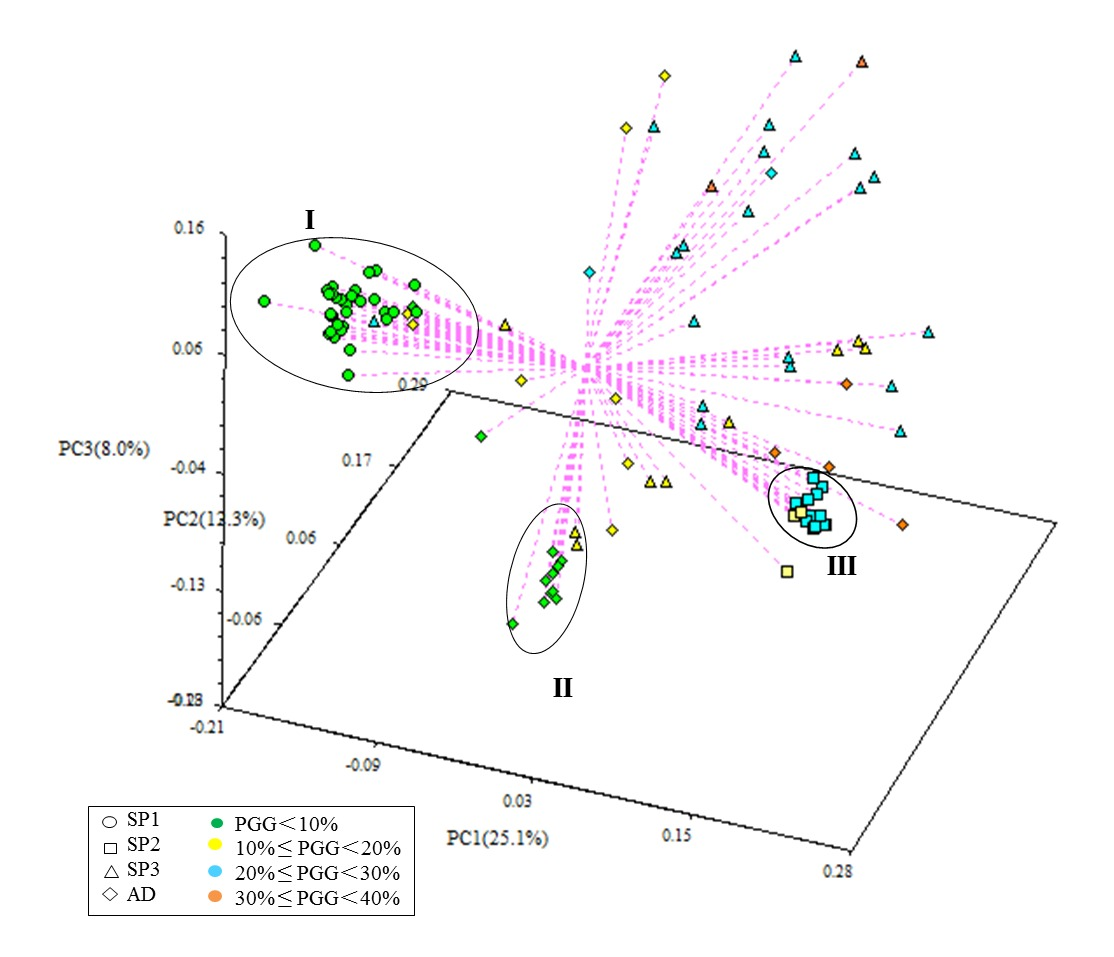

Supplement: S1 Fig — PC1, PC2 and PC3 refer to the first, second and third principal components, respectively. The numbers in parentheses refer to the proportion of the variance explained by the corresponding axes. Most ILs (47/48) with lower PGGs (< 10%) were clustered into two groups, Cluster I (37), Cluster II (10), while Cluster III contained 12 ILs with intermediate PGG (10% − 30%). The remaining 46 ILs with intermediate or higher PGG (10% − 40%) were distributed discretely. (TIF) [file pone.0161746.s001.tif]

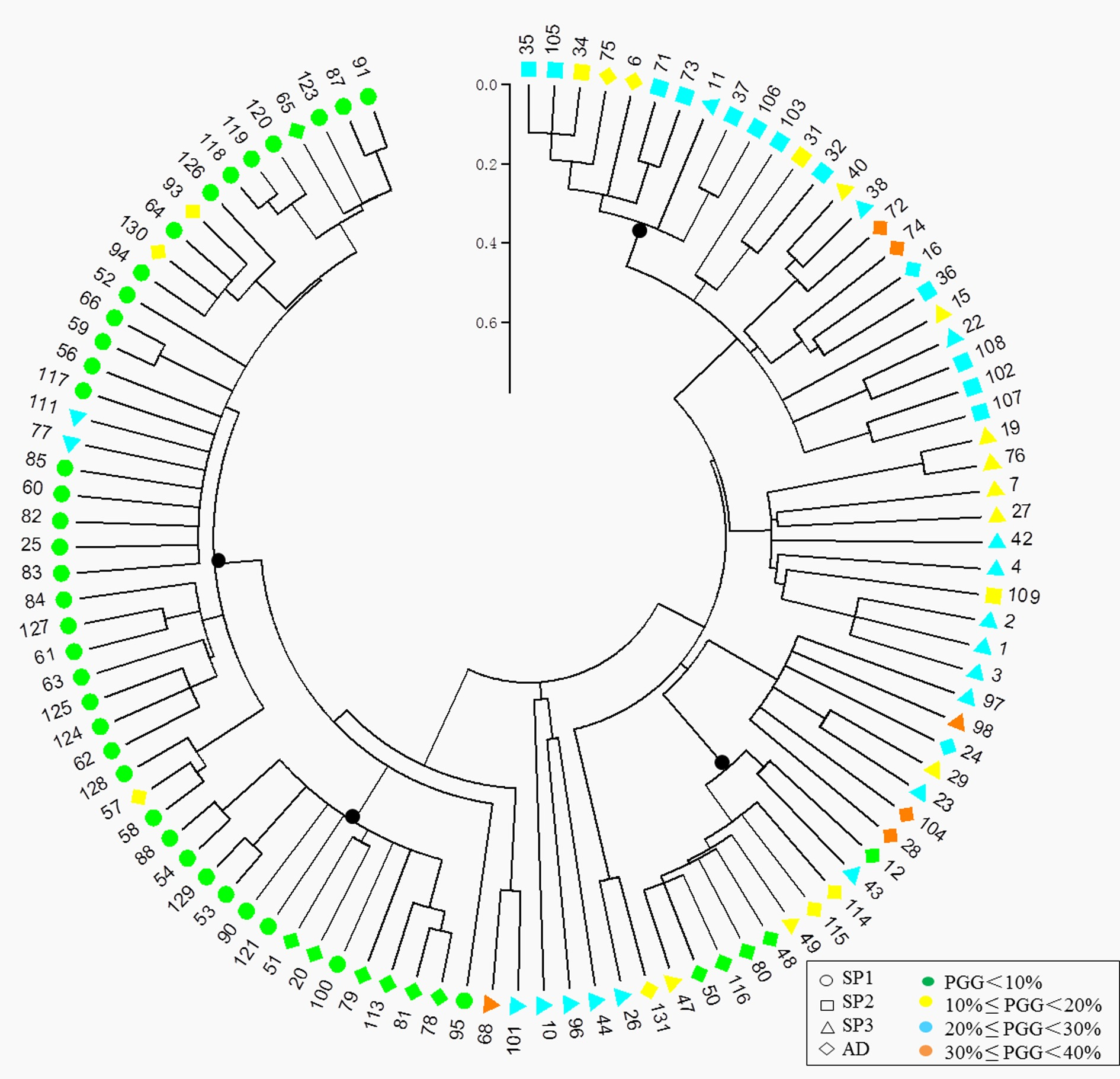

Supplement: S2 Fig — Most ILs (43/48) with lower PGG (< 10%) were assigned into two clusters on the phenotypic NJ tree. There were two smaller clusters. One contained ILs (9/10) with PGGs in the range 0% − 20% and another contained 10 ILs with PGGs in the range 10% − 30%. The remaining 38 ILs with PGGs (10% − 40%) were dispersed to different branches. (TIF) [file pone.0161746.s002.tif]

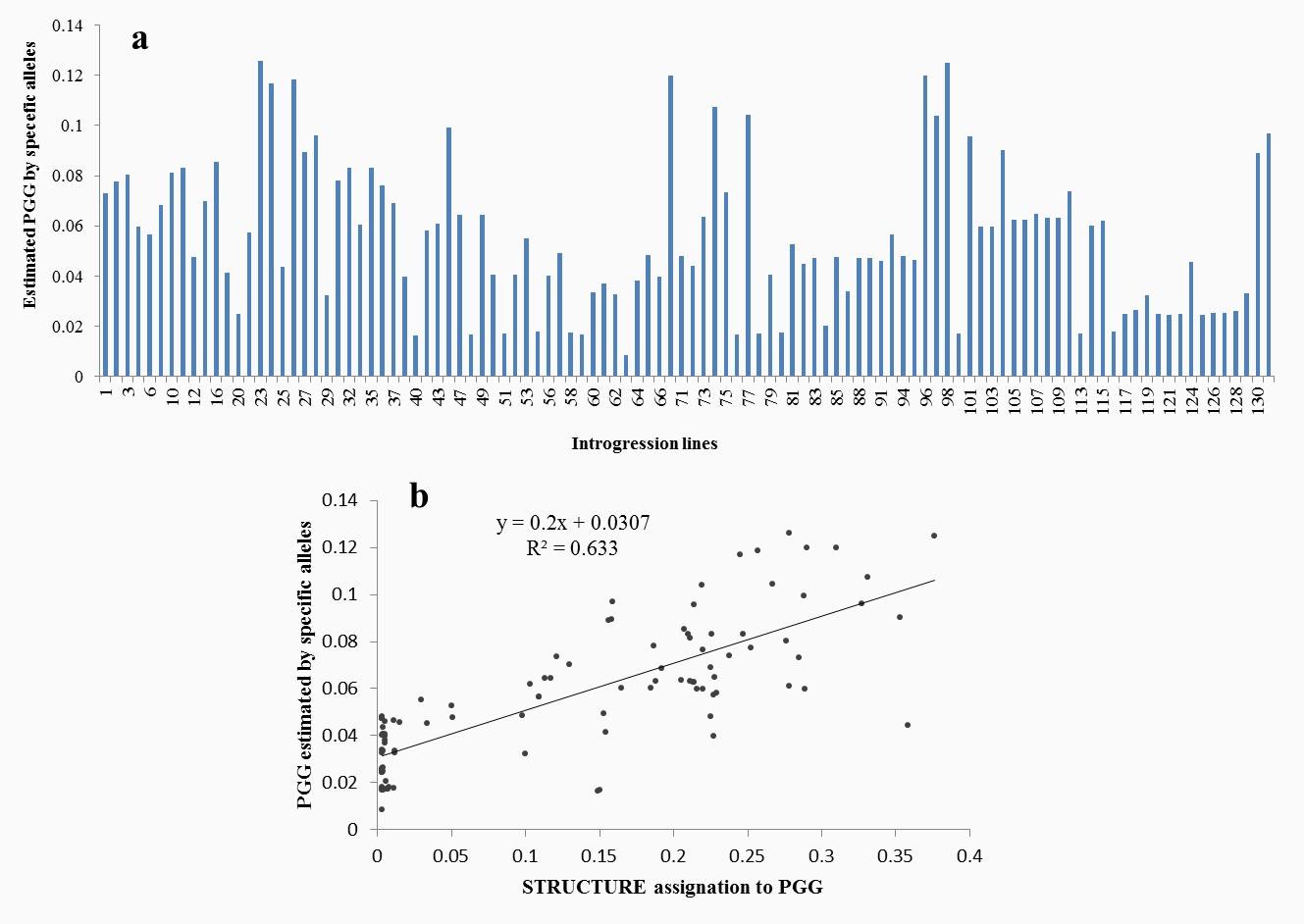

Supplement: S3 Fig — (a) PGG in the ILs estimated by using introgression alleles. (b) Comparison of PGG estimated by Bayesian method vs. PGG estimated by using introgression alleles in ILs. (TIF) [file pone.0161746.s003.tif]
