# Supplementary material for: Genetic Diversity and Phenotypic Variation in an Introgression Line Population Derived from an Interspecific Cross between Oryza glaberrima and Oryza sativa
Source: PLoS One. 2016 Sep 7;11(9):e0161746. doi: 10.1371/journal.pone.0161746 (PMC5014448; doi:10.1371/journal.pone.0161746)
Supplement: S1 Table — (DOCX) [file pone.0161746.s004.docx]

**S1 Table**. **Phenotypic and GGP variation between the 4 subpopulations**

| **Traits and PGG** | | **SP1** | **SP2** | **SP3** | **AD** |
| --- | --- | --- | --- | --- | --- |
| **PGG** | Mean | 0.01c | 0.21a | 0.22a | 0.12b |
|  | SD | 0.01 | 0.01 | 0.07 | 0.12 |
| **PH(cm)** | Mean | 116.46b | 110.73c | 116.83b | 121.00a |
|  | SD | 4.62 | 2.75 | 10.02 | 8.02 |
| **YP(g)** | Mean | 30.18b | 36.37a | 36.62a | 38.67a |
|  | SD | 5.26 | 5.84 | 7.12 | 5.70 |
| **TGW(g)** | Mean | 23.59a | 21.79c | 22.28bc | 23.21ab |
|  | SD | 0.93 | 0.64 | 3.17 | 1.44 |
| **SN(no/plant)** | Mean | 112.77c | 145.57a | 131.16b | 136.32b |
|  | SD | 11.87 | 16.33 | 21.31 | 13.06 |
| **FGP** | Mean | 0.78ab | 0.67c | 0.73b | 0.81a |
|  | SD | 0.05 | 0.08 | 0.09 | 0.09 |
| **PL(cm)** | Mean | 22.79c | 27.26a | 26.01b | 25.49b |
|  | SD | 0.78 | 0.81 | 2.58 | 1.66 |
| **PN(no/plant)** | Mean | 11.26b | 11.62ab | 12.63a | 12.37a |
|  | SD | 1.47 | 2.00 | 2.28 | 1.40 |
| **DF(d)** | Mean | 100.78a | 70.33d | 82.55c | 87.38b |
|  | SD | 1.81 | 1.18 | 9.64 | 10.38 |

PH = plant height; YP = yield per plant; TGW = thousand grain weight; SN = spikelet number per panicle; FGP = filled grain percentage; PL = panicle length; PN = panicle number per plant; DF = days to flowering
